# Treatment of *Leptothrix* Cells with Ultrapure Water Poses a Threat to Their Viability

**DOI:** 10.3390/biology4010050

**Published:** 2015-01-27

**Authors:** Tatsuki Kunoh, Tomoko Suzuki, Tomonori Shiraishi, Hitoshi Kunoh, Jun Takada

**Affiliations:** 1 Core Research for Evolutionary Science and Technology (CREST), Japan Science and Technology Agency (JST), Okayama 700-8530, Japan; E-Mails: tkunoh06@cc.okayama-u.ac.jp (T.K.); suzukito@cc.okayama-u.ac.jp (T.S.); hkunoh@cc.okayama-u.ac.jp (H.K.); 2 Graduate School of Natural Science and Technology, Okayama University, Okayama 700-8530, Japan; 3 Research Institute for Biological Sciences, Okayama 700-8530, Japan; E-Mail: tomoshir@bio-ribs.com

**Keywords:** *Leptothrix*, cell death induced by UPW, acidic pH to cell viability, lack of Ca^2+^ to cell viability, peptidoglycan, damage of bacterial membrane, relaxation of DNA, electrolytes leakage

## Abstract

The genus *Leptothrix*, a type of Fe/Mn-oxidizing bacteria, is characterized by its formation of an extracellular and microtubular sheath. Although almost all sheaths harvested from natural aquatic environments are hollow, a few chained bacterial cells are occasionally seen within some sheaths of young stage. We previously reported that sheaths of *Leptothrix* sp. strain OUMS1 cultured in artificial media became hollow with aging due to spontaneous autolysis within the sheaths. In this study, we investigated environmental conditions that lead the OUMS1 cells to die. Treatment of the cells with ultrapure water or acidic buffers (pH 6.0) caused autolysis of the cells. Under these conditions, the plasma membrane and cytoplasm of cells were drastically damaged, resulting in leakage of intracellular electrolytes and relaxation of genomic DNA. The autolysis was suppressed by the presence of Ca^2+^. The hydrolysis of peptidoglycan by the lysozyme treatment similarly caused autolysis of the cells and was suppressed also by the presence of Ca^2+^. However, it remains unclear whether the acidic pH-dependent autolysis is attributable to damage of peptidoglycan. It was observed that *L. discophora* strain SP-6 cells also underwent autolysis when suspended in ultrapure water; it is however, uncertain whether this phenomenon is common among other members of the genus *Leptothrix*.

## 1. Introduction

A type of aquatic Fe/Mn-oxidizing bacteria, the genus *Leptothrix*, is characterized by its formation of an extracellular, microtubular, Fe-encrusted sheath [1,2,3,4,5]. When isolated strains are cultured in artificial media containing a Fe source, most sheaths envelop bacterial cells at an early stage of incubation. However, the majority of sheaths, especially those that look thick and yellow-brownish, become at least partially empty thereafter [1], resembling most of the aged sheaths of *L. ochracea* harvested from natural aquatic environments. Emerson *et al.* [3] noted that only *ca.* 10% of the sheaths contained chained bacterial cells at their actively growing stage. According to Ghiorse [4], *L. ochracea* forms and vacates Fe-encrusted sheaths very rapidly in low-nutrient, Fe-containing enrichment culture, leaving a large mass of empty sheaths. We previously reported that aged sheaths produced by *Leptothrix* sp. strain OUMS1 (NITE BP-860, hereafter, referred to as OUMS1) in culture also became hollow due to spontaneous autolysis of the cells within the sheaths [1]. However, it remains unsolved why and how *Leptothrix* cells die in the sheaths. The internal transcribed spacer (ITS) analysis of ribosome DNA revealed that OUMS1 was close to a known species *L. cholodnii* with only a difference of two base pairs [6].

The self-destructive end stage of the bacterial life cycle, termed autolysis, has been studied over 100 years. Bacterial autolysis is influenced by a variety of factors including growth phase, environmental pH, osmotic pressure, temperature as well as availability of oxygen, nitrogen, and carbon source [7]. Various methods for inducing bacterial autolysis were strenuously investigated against *Escherichia coli* cells [8,9]. The treatment of *E. coli* cells with antibiotics such as d-cycloserine, β-lactams, and moenomycin efficiently blocked peptidoglycan synthesis to result in bursting of the cells under a hypotonic environment due to damage of the plasma membrane [9]. According to Leduc and van Heliencort [8], the osmotic shock, comprised of a quick downshock with distilled water before an upshock with 0.5 M sodium acetate (pH 6.5), readily drives *E. coli* cells to autolytic death. Intriguingly, the presence of Mg^2+^ or Ca^2+^ prevents this osmotic shock-dependent autolysis. Similar effects of these divalent cations on inhibiting autolysis were reported in several other Gram-negative bacteria [10,11]. Although the action of these cations is poorly understood, Rayman and Macleod [11] noted that the interaction of Mg^2+^ or Ca^2+^ with the peptidoglycan layer holds a key to prevent autolysis of marine pseudomonads.

Referring to these earlier reports, we assumed that analysis of the responses of OUMS1 cells in unusual environmental conditions might provide some insight into the mechanism of spontaneous autolysis of *Leptothrix* in sheaths and their hollowing mechanism. To approach these mechanisms more closely, we undertook to examine basic physiology of OUMS1 cells affected by the environmental conditions which promoted autolytic death of the cells, and in particular, the effects of ultrapure water on the sudden environment changes which might affect the bacterial viability.

## 2. Experimental Section

### 2.1. Strains, Medium, Culturing and Water

OUMS1 cells isolated from flocculent ocherous deposits in a biological freshwater purification plant in Joyo City, Kyoto Prefecture, Japan [6] and *L. discophora* strain SP-6 (ATCC 51168) were employed in this study. Precultures of OUMS1 and SP-6 cells were recovered from frozen stock and separately streaked on the silicon–glucose–peptone agar medium (pH 7.0) (for its components, see Table 1) (hereafter, referred to as SGP7.0; means a liquid medium unless otherwise noted) devised by Sawayama *et al.* [6] and incubated at 20 °C for seven days. Single colonies were independently picked with autoclaved toothpicks, transferred to 25 mL of SGP7.0 medium in 50 mL conical tubes (BD Bioscience, Bedford, MA, USA), and incubated on a rotary shaker (EYELA FMC-1000, Tokyo Rikakikai, Tokyo, Japan) at 20 °C and 70 rpm [6]. After 2–3 day incubation, 1 mL of the cell suspension (adjusted to 10 cfu/mL by densitometry (Nanodrop 2000C, Thermo Fisher Scientific Inc., Waltham, MA, USA)) was transferred to 100 mL of SGP7.0 in glass flasks, followed by incubation for an additional 2–3 days.

Ultrapure water (hereafter, referred to as UPW) was prepared from tap water using the MilliQ Direct-Q/UV (EMD Millipore, Billerica, MA, USA) and employed for the UPW-treatment after autoclaving. Ground water obtained from the Okayama University Research Farm was membrane-sterilized by using the 0.22 μm-syringe filter (Supelco, Bellefonte, PA, USA.) immediately before use (hereafter, referred to as GW).

**Table 1:** Composition of SGP7.0.

Component	Amount (g/L)	Concentration (mM)
Glucose	1.000	5.55
Soy peptone	1.000	ND
Na_2_SiO_3_∙9H_2_O	0.200	0.70
CaCl_2_∙2H_2_O	0.044	0.30
MgSO_4_∙7H_2_O	0.041	0.17
Na_2_HPO_4_∙12H_2_O	0.076	0.21
KH_2_PO_4_∙2H_2_O	0.020	0.15
HEPES	2.380	10.00

Medium was adjusted to pH 7.0 with 0.1N NaOH, then brought to 1 liter with UPW. ND: not determined.

### 2.2. Drop- and Cfu-Tests to Examine Growth of Cells

Exponentially growing cells cultured in 100–500 mL of SGP7.0 for two days were harvested, washed twice in UPW, 10 mM Tris-HCl buffer, 10 mM *N*-2-hydroxyethylpiperazine-*N*’-2-ethanesulfonic acid (HEPES) buffer, or SGP7.0 (hereafter, all referred to as suspension agent) by centrifugation (2400 X *g*, 5 min). These cell suspensions were incubated in the same respective suspension agents at room temperature for 1.5 or 3 h. A subset of all of the cell suspensions (3 mL) was taken and washed twice in SGP7.0 by centrifugation (2400 X *g*, 5 min) (hereafter, referred to as washed cell suspension). The subset was diluted serially 10-fold when necessary and drop-inoculated to a SGP7.0 agar plate, followed by incubation for several days at 20 °C before examining colony formation (hereafter, referred to as drop-test). In addition, for quantitative evaluation of the cell growth, the washed cell suspensions were diluted serially 10-fold with SGP7.0 and immediately inoculated onto SGP7.0 agar plates. After 4 days incubation, the colony-forming unit (cfu) was counted in six replicates. The average cfu numbers were recorded (hereafter, referred to as cfu-test).

When necessary, the additives such as the respective SGP7.0 components, inorganic chemicals or enzymes were dissolved in the proper suspension agents and subjected to the tests of interference of these additives with autolysis as described later (Figure 3B–F, see Supplementary Figure S1A,E). Alternatively, 0.05–0.5 mM ethylene glycol tetraacetic acid (EGTA), frequently used as a Ca^2+^ selective chelator [12], was added to SGP7.0 to reduce or eliminate Ca^2+^ from peptone, one of the SGP components, and their autolysis-inducing effects were examined.

### 2.3. LIVE/DEAD Stain to Examine Viability of Cells

After the washed cells were kept in the respective suspension agents at room temperature for 0, 1.5, or 3 h, viability of the cells was examined using the LIVE/DEAD BacLight Bacterial Viability Kit (Life Technologies, Carlsbad, CA, USA) [1]. Briefly, the component B (1.67 mM SYTO9 dye and 18.3 mM propidium iodide (PI)) was added to the cell suspensions at a 1:300 dilution and the reaction mixture was kept at room temperature for 20–30 min. Differential interference contrast (DIC) and fluorescence images of stained cells were observed with the BX51 System Microscope (OLYMPUS, Tokyo, Japan) equipped with a U-MWIB3 dichroic mirror unit (460–490 nm excitation filter and 520 nm emission wavelengths) (hereafter, referred to as L/D stain). By this method, viable and dead cells were effectively distinguished by staining with two different DNA-binding dyes, SYTO9 and PI. SYTO9 is permeable through the intact plasma membrane, giving the live cells greenish fluorescence, while PI can stain only membrane-damaged cells, giving the dead cells reddish fluorescence. Yellowish orange (a mixture of reddish and greenish) fluorescence occasionally occurred when the cells of halfway-damaged plasma membrane were stained [13].

### 2.4. Measurement of pH, Conductivity, Osmotic Pressure, and Ion Concentration in Suspension Agents or Cell Suspensions

To know basic status of the respective suspension agents used in this study, their pH and conductivity were measured using the LAQUAtwin water quality meters (Horiba, Kyoto, Japan) and osmotic pressure using OSMOTRON-5 (Orion Riken, Tokyo, Japan), respectively. To examine leakage of intracellular electrolytes from autolytic cells, conductivity and K^+^/Ca^2+^ concentrations were monitored in the cell suspensions, as follows [14]: Exponentially growing cells were collected and washed twice with 10 mM HEPES (pH 7.0) (hereafter, HEPES7.0) by centrifugation (2400 X *g*, 5 min). The resultant cell pellets were suspended in 35–45 mL of HEPES7.0, HEPES6.0 containing 3 mM CaCl_2_, UPW, or 200 μg/mL of lysozyme (Sigma-Aldrich, St Louis, MO, USA). Every 30 min after the onset of suspension, a subset of the respective cell suspensions (3 mL) was passed through 0.2 μm-syringe filter (Supelco) to remove the cells and debris, followed by measurement of conductivity and cation concentration using the LAQUAtwin water quality meters (Horiba, Kyoto, Japan). Because 0.1 N NaOH was used for adjustment of pH, the initial conductivity of HEPES7.0 suspension was inevitably higher than that of UPW- and 6.0-suspensions, as explained in Figure 5B,E.

### 2.5. Isolation of Genomic DNAs from OUMS1 Cells

The procedure was performed as described previously [6] with slight modifications. Exponentially growing OUMS1 cells in 500 mL culture were collected by centrifugation at 3600 X *g* for 5 min and the resultant cell pellet was resuspended in 15 mL of SGP7.0. A 1/4 aliquot of the cell suspension was washed twice in SPG7.0, SGP6.0, HEPES7.0, or HEPES6.0 by centrifugation 2400 X *g* for 5 min and stood in the respective washing agents at room temperature. After 3 h, these cells were washed in Tris-HCl/EDTA buffer (10 mM Tris-HCl and1 mM EDTA, pH 8.0) (hereafter, TE buffer) by centrifugation and suspended in 5 mL of TE buffer containing 20 μg/mL Proteinase K (Takara Bio Inc., Shiga, Japan) and 0.6% sodium dodecyl sulfate (SDS, Nacalai Tesque, Kyoto, Japan). After incubation at 65 °C for 1 h, 1 mL of 5 M NaCl and 800 μL of CTAB/NaCl solution (10% cetyl trimethyl ammonium bromide and 0.7 M NaCl) were added to the cell suspension, and incubated at 65 °C for an additional 1 h. To eliminate proteins, the cell suspension was sequentially extracted using an equal volume of chloroform:isoamyl alcohol (24:1), phenol:chloroform:isoamyl alcohol (25:24:1), and chloroform. For precipitation of nucleic acids, the resultant supernatant was added to an equal volume of isopropanol. After the reaction mixture was kept at −20 °C for 1 h, the pellet was collected by centrifugation (20,000 X *g*, 15 min), followed by rinsing in 70% ethanol. After drying, the pellet was suspended in 500 μL of TE buffer containing 20 μg/mL Ribonuclease A (Takara Bio Inc., Shiga, Japan) and incubated at 37°C for 30 min, followed by DNA extraction using phenol:chloroform:isoamyl alcohol (25:24:1) and chloroform. The ethanol-precipitated genomic DNAs were dissolved in TE buffer and loaded on 1% agarose gel. The gel was stained by the GelRed Nucleic Acid Gel Stain (Biotium Inc., Hayward, CA, USA) after electrocataphoresis. DNA bands were visualized using the UV illuminator (Dolphin-View2) (Kurabo, Osaka, Japan).

### 2.6. Electron Microscopy

The sample preparation was performed as described previously [1]. Briefly, specimens were collected by centrifugation and fixed with a mixture of 2.5% glutaraldehyde, 1% OsO4, and 4.5% sucrose in 100 mM cacodylate buffer (pH 7.0) on ice for 2 h and then embedded in 3% agar. Small pieces of the agar block were dehydrated in a graded series of ethanol and embedded in Quetol 651 resin mixture (Nisshin EM, Tokyo, Japan). Ultrathin sections were stained with uranyl acetate and lead solutions and observed with a transmission electron microscope (H-7500, Hitachi, Tokyo, Japan) operated at an accelerating voltage of 80 kV.

## 3. Results and Discussion

### 3.1. Autolysis of OUMS1 Cells Caused by Prolonged Treatment with UPW

**Figure 1:**
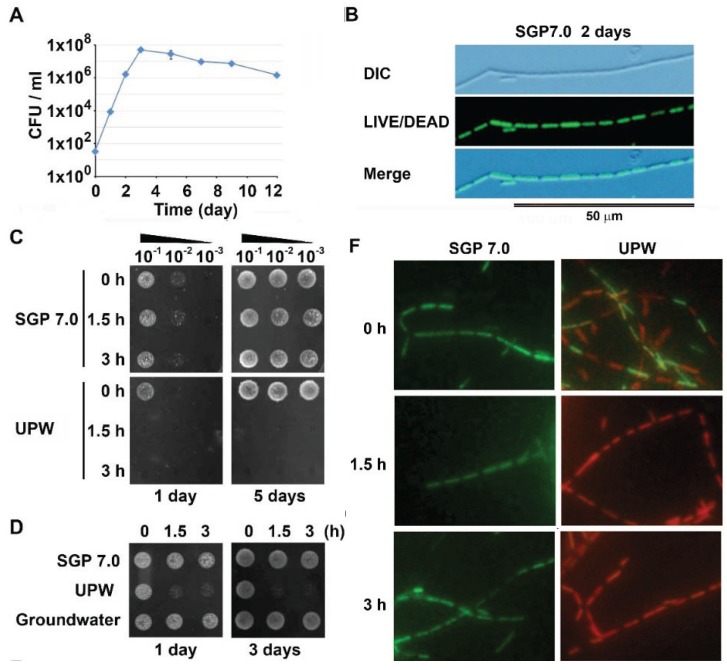

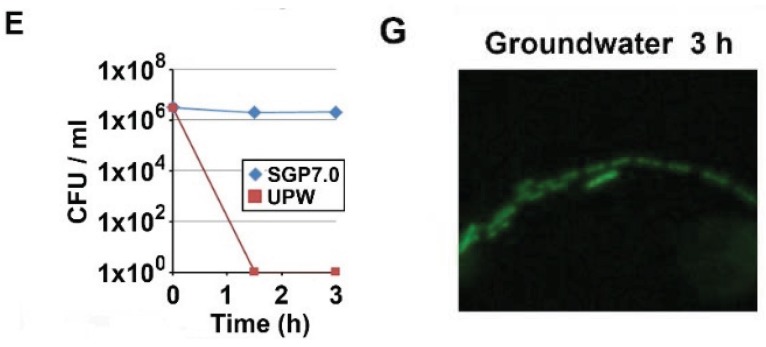
Autolysis of OUMS1 cells caused by UPW treatment. The cells were harvested from 2-day culture in SGP 7.0 prior to the treatment. (**A**) Time course of growth of OUMS1 cells in SGP7.0 determined by cfu-test. (**B**) Top panel: a DIC image of chained cells enveloped a thin sheath in SGP7.0. Middle panel: L/D stain image of the same chained living cells. Bottom panel: A merged image of the above DIC and L/D stain images. (**C**) Colony formation by OUMS1 cells treated with SGP7.0 or UPW for 0–3 h (drop test). Note no colony formation in the 1.5 or 3 h UPW treatment. (**D**) Colony formation by OUMS1 cells treated with SGP7.0, UPW, or GW. Note colony formation in GW and SGP7.0 treatments but no formation in UPW treatment. (**E**) The population of living cells in the suspension in SPG7.0 or UPW (cfu-test). Note that the living cell population reached zero in UPW by 1.5 h. (**F**) The L/D images of the cells incubated in SPG7.0 or UPW for 0–3 h. Note that all cells were alive in SGP7.0 treatment but some cells were dead already at 0 h UPW treatment. (**G**) The L/D image of the living cells treated with GW for 3 h.

The cfu-test revealed that in SGP7.0, OUMS1 cells exponentially grew for three days after inoculation and thereafter their growth reduced day by day (Figure 1A). Almost all chained cells were demonstrated to be alive by the L/D stain during the exponential phase. In the merged image with the DIC image, thin microtubular sheaths were distinguished around the chained cells (Figure 1B). Leduc and van Heliencort [8] reported that *E. coli* cells lost their viability when washed with distilled water prior to the 0.5 M sodium acetate (pH 6.5). Referring their method, exponentially growing OUMS1 cells were washed twice with UPW by centrifugation for 5 min (hereafter, UPW-washed inoculum) prior to the drop-test. In control, the cells were washed in SGP7.0 in a similar manner and subjected to the drop-test. As illustrated in Figure 1C (upper line), both inocula formed colonies within one day after the onset of incubation, depending on dilution of cell suspensions. After 5 day incubation, colony was formed when the washed inoculum was suspended in SGP7.0 regardless of dilution of cell suspension prior to drop-test. By contrast, only when the washed suspension in UPW was subjected to drop-test immediately after washing, colony formation occurred after 5 day incubation regardless of dilution of the cell suspension (Figure 1C, UPW top line). These results suggested that the initial double UPW washing (total 10 min) prior to the drop-test might not affect viability of the cells. Therefore, we tentatively defined this stage as the 0 h-treatment. The cells suspended in UPW for 1.5 or 3 h after the double washing never formed colonies, although those suspended in SGP7.0 for the same period did (Figure 1C, middle and bottom lines). The cells suspended in GW for the same period preserved viability, as expected (Figure 1D, bottom line). These results apparently demonstrated that the prolonged treatment with UPW could kill the cells but the same treatment with GW did not.

The subsequent cfu-test revealed that the cell population was 3.2 × 10^6^ and 3.1 × 10^6^ cfu/mL in SGP7.0 and UPW at 0 h-treatment, respectively. As shown in Figure 1E, the cell population in SGP7.0 was almost steady until 3 h after the onset of treatment, while that in UPW reached zero by 1.5 h.

To verify the reliability of the drop- and cfu-tests, the L/D stain was performed against the cell suspensions in SGP7.0, UPW, and GW. In SGP7.0 or GW, almost all cells gave greenish fluorescence (alive state) irrespective of the length of treatment period (Figure 1F,G). In 0 h-treatment with UPW, approximately half of the cells fluoresced greenish, while the rest of cells fluoresced reddish (Figure 1F) and then, all cells fluoresced reddish in 1.5 and 3 h treatments (Figure 1F). These results indicate that approximately half of the cells died during the initial double UPW washing. Thus, it is most likely that colony formation in 0 h-treatment with UPW, which was seen in drop- and cfu-tests, is attributable to growth of cells which survived even after the initial double UPW washing. Therefore, for judging viability of the cells, the drop- and cfu-tests are reliable only when colony formation is negative, because if some living cells exist after treatment, they are able to form colonies during incubation for these tests. With repeated careful experiments, we found that the degree of incidence of dead cells in various treatments was largely dependent on the numbers of cells in washed suspension prior to the L/D stain. When the cell number in UPW-washed inoculum was adjusted to approximately 1/5 cfu/mL prior to incubation in UPW, all cells were killed to give reddish fluorescent response in the L/D stain, resulting in the negative responses in both drop- and cfu-tests. Therefore, the suspension with reduced number of cells was used for some experiments where the death of all cells was required, as described later (Figure 3C,D).

### 3.2. Acidic pH to Induce Autolysis of the Cells

**Figure 2:**
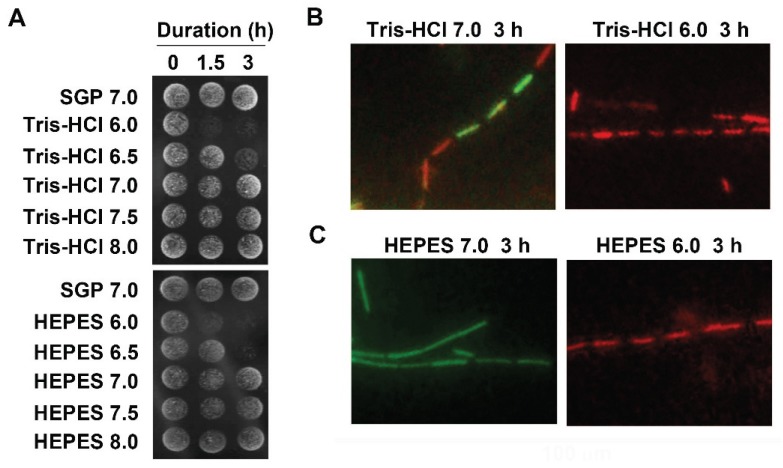
Effects of pH on induction of autolysis of OUMS1 cells. (**A**) Colony formation of OUMS1 cells treated with 10 mM Tris-HCl or HEPES buffers adjusted to pH 6.0–8.0 for 0–3 h, determined by drop test (3-day incubation). (**B**), (**C**) L/D stain of OUMS1 cells treated with 10 mM Tris-HCl or HEPES buffers (pH 6.0 and 7.0) for 3 h. Note that some cells died in Tris-HCl buffer even at pH 7.0 but not in HEPES7.0.

Most bacteria have mechanisms that maintain their internal, cytoplasmic pH within a narrower range than the pH outside the cell, termed “pH homeostasis” [15]. As mentioned above, Leduc and Heliencort [8] reported that the osmotic shock gave rise to significant decreases in turbidity and viability of *E. coli* cells. Conversely, Wegner *et al.* [10] reported that *Neisseria gonorrhoeae* readily underwent autolysis when suspended in HEPES buffer at alkaline pH values. These reports suggest that pH might be one of the key factors to induce bacterial autolysis. Considering pH values of SGP7.0, UPW, and GW which were used in the above experiments (7.0, 5.7, and 6.6, respectively) (Table 2), it seemed likely that acidic pH might be one of the environmental factors to kill OUMS1 cells, unlike the case of *N. gonorrhoeae*. To examine this possibility, cultured OUMS1 cells were washed with 10 mM Tris-HCl or HEPES buffer (pH 6.0–8.0) and suspended in the respective same buffers. As shown in Figure 2A, the cells formed colonies in 0 h treatments with all pH levels of both buffers. However, the 1.5 or 3 h prolonged pH 6.0 treatment using both buffers never allowed the cells to form colonies. By contrast, the cells suspended in both buffers (pH 7.0–8.0) developed colonies similarly as in SGP7.0, regardless of the length of treatment period. In accordance with these results, the L/D stain revealed that all cells treated with Tris-HCl6.0 or HEPES6.0 were dead (Figure 2B,C). These results strongly suggested that OUMS1 cells might autolyze when encountered with acidic pH 6.0. As evident from the L/D stain images some cells died even at pH 7.0 of Tris-HCl (Figure 2B) but all cells survived at pH 7.0 of HEPES buffer (Figure 2C), suggesting that the former buffer may be somehow harmful to OUMS1 cells. Again, Leduc and Van Heliencort’s conclusion [8] concerning the osmotic shock to kill *E. coli* cells led us to suspect that low and/or conductivity of our suspension agents might induce autolysis of OUMS1 cells. As expected, UPW had extremely low conductivity and zero osmotic pressure (Table 2). However, Tris-HCl6.0, which induced autolysis had much higher conductivity and osmotic pressure than those of GW which never induced autolysis (Table 2). Accordingly, in the case of OUMS1, acidic pH is a cause to trigger autolysis rather than osmolality and/or conductivity, unlike in *E. coli* [8].

**Table 2:** Solutions and media used in the experiments and measured properties.

Solution	pH	Conductivity (mS/cm)	Osmotic pressure (mosmol/kg)
Tap water	7.1	0.096	0.33
UPW (autoclaved)	5.7	0.002	0.00
10 mM Tris-HCl 6.0	6.0	0.850	18.33
10 mM Tris-HCl 6.5	6.4	0.890	18.00
10 mM Tris-HCl 7.0	6.9	0.870	18.00
10 mM HEPES 6.0	6.0	0.019	14.00
10 mM HEPES 6.5	6.5	0.054	14.00
10 mM HEPES 7.0	7.0	0.131	13.67
Groundwater	6.6	0.350	10.00
Groundwater (filtrated)	6.6	0.360	10.33
SGP 6.0	6.0	0.510	31.00
SGP 6.5	6.5	0.480	31.33
SGP 7.0	7.0	0.510	31.33

### 3.3. Suppression of Autolysis of OUMS1 Cells by Ca^2+^

**Figure 3:**
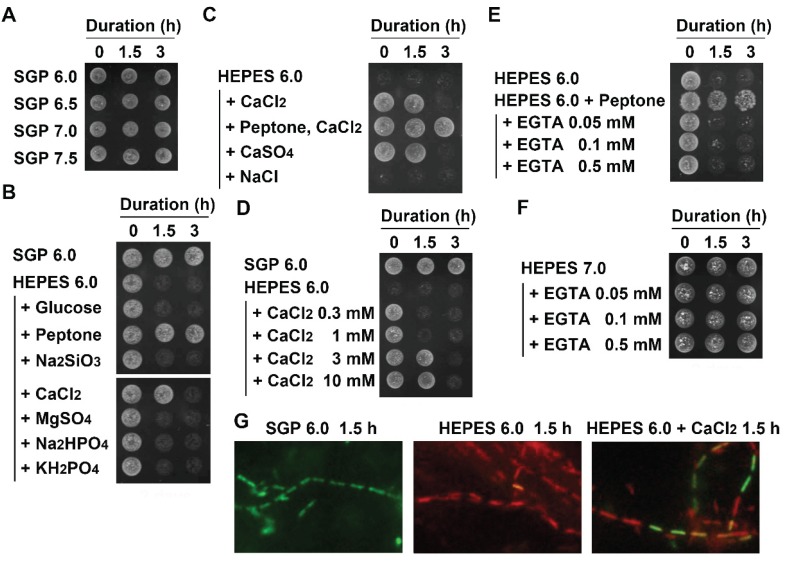
Ca^2+^ to suppress the acidic pH-inducing autolysis of OUMS1. (**A**) Colony formation of OUMS1 cells treated with SGP regardless of pH 6.0–7.5, determined by 2 day-incubation of drop-test. (**B**) Effects of the respective components of SGP to suppress autolysis of the cells treated with HEPES6.0, determined by 2 day-incubation of drop-test. Note that peptone and CaCl_2_ succeeded in suppressing the autolysis but other components did not. (**C**) The HEPES6.0-inducing autolysis suppressed by the presence of Ca^2+^ but not by Cl^−^. (**D**) Suppression of HEPES6.0-inducing autolysis by higher concentrations of CaCl_2_. (**E**) HEPES6.0-inducing autolysis not suppressed by removal of Ca^2+^ from peptone. (**F**) No interference of 0.05–0.5 mM EGTA with colony formation of the cells. (**G**) The L/D stain showing alive and dead cells caused by 1.5 h treatment of SGP6.0, HEPES6.0, or HEPES6.0 plus CaCl_2_.

Autolysis of *E. coli*, *N. gonorrhoeae*, and *Pseudomonas* sp. was reported as suppressed or prevented by divalent cations such as Ca^2+^ and Mg^2+^ [8,10,11]. Although it was concluded that acidic pH 6.0 held a key to induce autolysis of OUMS1 cells, we still wondered whether acidic pH was only one factor to induce autolysis. Thus, OUMS1 cells precultured in SGP7.0 were treated with pH-adjusted SGP (6.0–7.5) (hereafter, indicated as SGP6.0-SGP7.5) for 1.5 or 3 h (Figure 3A). Unexpectedly, even 3 h treatment of SGP6.0 did not kill the cells, suggesting that acidic pH may not be only one factor to induce autolysis and that some components of SGP could contribute to suppress autolysis. Among the respective components of SGP (concentrations in Table 1) that were dissolved in HEPES6.0 independently, only soy peptone or CaCl_2_ suppressed autolysis, while MgSO_4_, Na_2_HPO_4_, and KH_2_PO_4_ failed to do so (Figure 3B). According to Wegener *et al.* [10] The HEPES (pH 8.5)-autolysis of *N. gonorrhoeae* cells was prevented by divalent cations such as Mg^2+^, Ca^2+^, Mn^2+^, and Ba^2+^ but monovalent cations such as Na^+^, K^+^, and Li^+^ did not show remarkable effects. Similarly, Rayman and Macleod [11] found that Mg^2+^ prevented autolysis of marine pseudomonads cells but Na^+^ did not. The present results showed that Mg^2+^ was ineffective to prevent HEPES6.0-inducing autolysis of OUMS1 cells in contrast to the case of *N. gonorrhoeae* cells. Therefore, it seems likely that interference of divalent cations with autolysis is variable among bacterial species.

As mentioned above, the incidence of dead cells in various treatments is largely affected by cell numbers in suspension before the treatments. Thus, a lower number of the cells (approximately 1/5 of the previous test (Figure 3B)) was employed to examine the suppressive effects of Ca^2+^ and peptone for autolysis. In this experiment (Figure 3C,D), colonies were not formed even in 0 h-treatment of HEPES6.0 unlike the previous results (Figure 2A and Figure 3B), indicating that all cells were killed during the initial double washing before 0 h-treatment. It was confirmed again that CaCl_2_, CaSO_4_, and peptone had an entirely similar suppressive effect against autolysis (though addition of peptone was more effective than single addition of Ca^2+^) but NaCl did not (Figure 3C), suggesting that Ca^2+^ could be important but the anion, Cl^−^, may not in suppression of autolysis. Subsequently, OUMS1 cells were treated with HEPES6.0 containing 0.3–10 mM CaCl_2_ for 1.5 or 3 h prior to the drop test. Results indicated that 3 and 10 mM had the suppressive effect in 1.5 h treatment but this effect did not last for 3 h (Figure 3D). Reduction or removal of Ca^2+^ from peptone with varied concentrations of EGTA was successful in inducing autolysis (Figure 3E). Colonies formed when the cells were treated with HEPES7.0 containing 0.05–0.5 mM EGTA (Figure 3F), indicating that EGTA, itself, did not suppress the cell growth. However, concerning this EGTA effect on the cell growth, it was demonstrated by Holland *et al.* [12] that some strains displayed reduced growth and inhibition of cell division in the presence of EGTA during culturing. The L/D stain supported results of the above drop tests (Figure 3G): all cells fluoresced greenish in SGP6.0, while reddish in HEPES6.0. As coincident with Figure 3D, some cells were greenish and others reddish after 1.5 h treatment with HEPES6.0 plus 3 mM CaCl_2_. All these results indicate that acidic pH was a major cause of autolysis of OUMS1, and that Ca^2+^ could be a suppressor of the pH-dependent autolysis. Since Ca^2+^ is an essential ion to maintain cell structure, motility, transport and cell differentiation in prokaryotes as well as eukaryotes [16,17,18,19], lack of Ca^2+^ must be fatal for OUMS1 when treated with UPW.

### 3.4. Damage of Plasma Membrane and Cytoplasm in UPW- and HEPES6.0-Treated Cells

**Figure 4:**
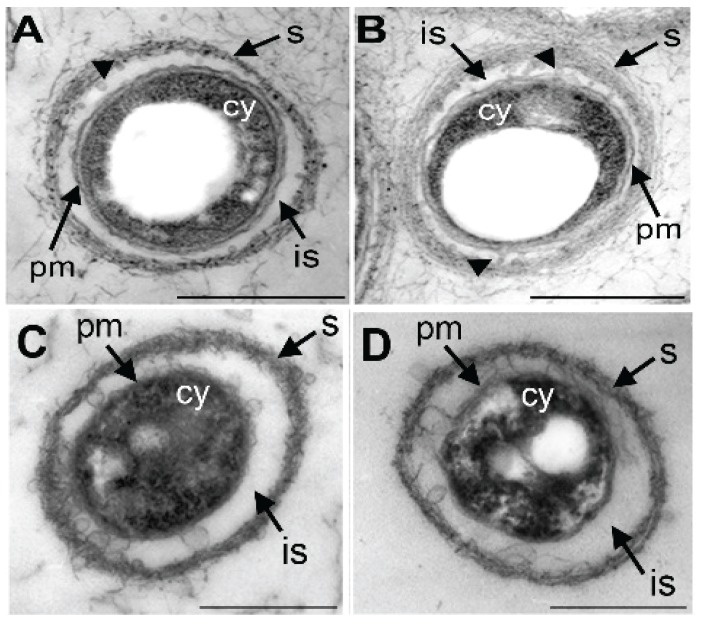

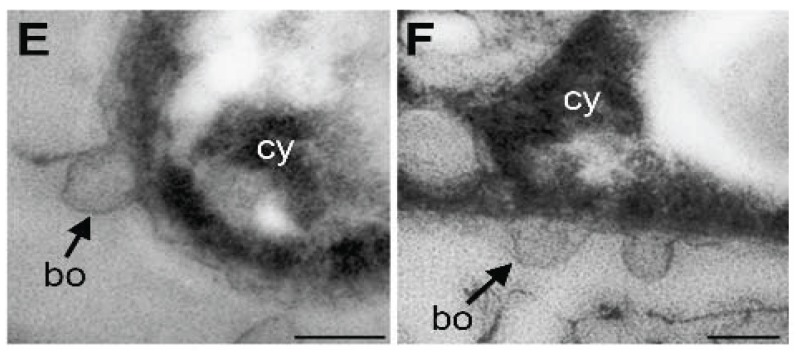
Figure 4. Transmission electron microscopy images of the cells treated with SGP7.0, HEPES7.0, UPW, or HEPES6.0 for 3 h. (**A**,**B**) In SGP7.0 (**A**) and HEPES7.0 treatments (**B**) the cells having double layered-plasma membrane (pm) and normal-looking cytoplasm (cy). The nearly parallel-arrayed fibrous sheath (s) formed across the intervening space (is). Globular secretion bodies seen in the intervening space in places (arrowheads). (**C**,**D**) In UPW (**C**) and HEPES6.0 treatments (**D**) the electron-dense coagulated cytoplasm (cy) surrounded hardly indistinguishable plasma membrane (pm) and electron-dense disordered sheath (s). (**E**,**F**) In UPW (**E**) and HEPES6.0 treatments (**F**) bulbous outgrowth (bo) of outer plasma membrane. Scale bar = 500 nm in (**A**–**D**) and 100 nm in (**E**,**F**).

The normal-looking cytoplasm of live cells treated with SGP7.0 or HEPES7.0 for 3 h was enveloped with a double-layered plasma membrane (Figure 4A,B). Furutani *et al.* [19] demonstrated TEM images of globular and/or thread-like secretion bodies arising from the outer plasma membrane of OUMS1 cells. Similar secretion bodies were observed on the surface of SGP7.0- or HEPES7.0-teated cells (Figure 4A,B, arrowheads). As Furutani *et al.* [19] illustrated, the cells were encompassed with a thin initial sheath across the intervening space from the cell (Figure 4A,B). This initial sheath was comprised of parallel-arranged fibrils. In contrast, the dead cells caused by 3 h treatment with HEPES6.0 and UPW had electron-dense coagulated cytoplasm enveloped by a poorly distinguishable electron-dense plasma membrane (Figure 4C,D). Notably, bulbous outgrowths arose from the outer plasma membrane in places (Figure 4E,F) and the initial sheath turned more electron dense with disordered fibrous structures. These observations apparently show that UPW and HEPES6.0 treatments damaged structure and probably function of plasma membrane and cytoplasm of OUMS1 cells.

### 3.5. Relaxation of Genomic DNA and Leaking of Electrolytes Caused by Autolysis-Inducing Treatments

**Figure 5:**
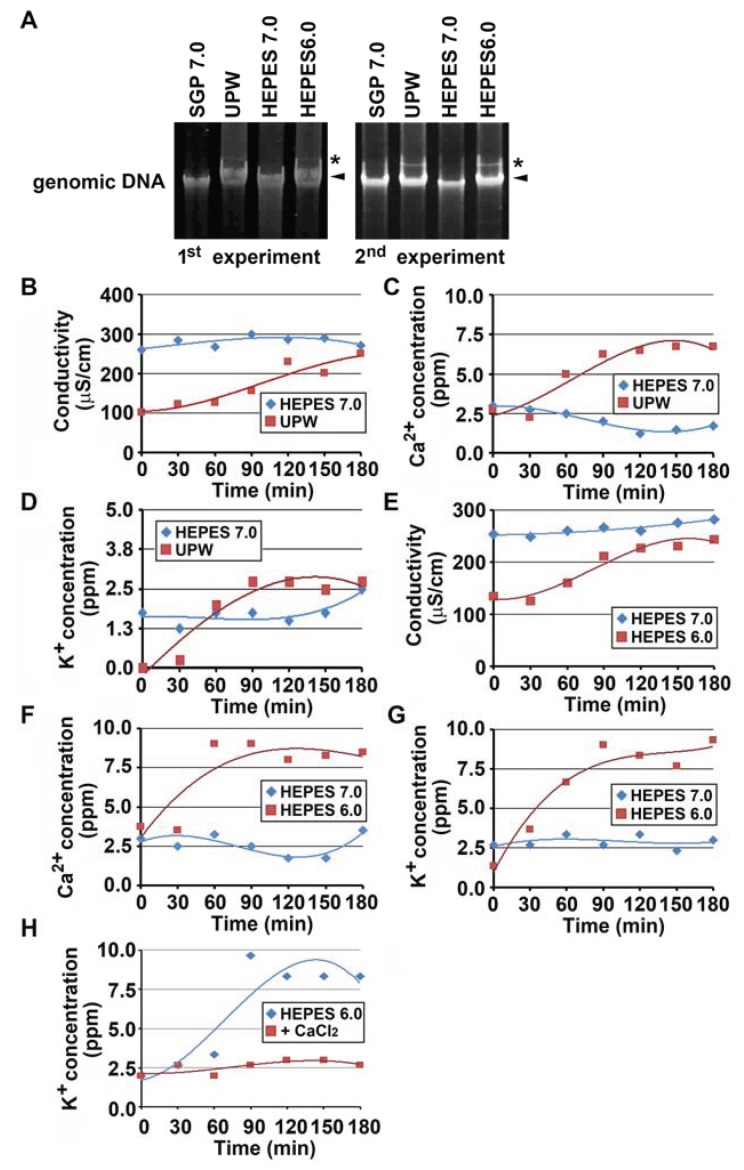
Relaxation of genomic DNA and electrolytes loss in OUMS1 cells treated with SGP7.0, HEPES7.0, HEPES6.0, or UPW for 3 h. (**A**) Genomic DNAs isolated from the cells treated with SGP7.0, UPW, HEPES7.0 or HEPES6.0 for 3 h. Arrowheads and asterisks indicate major and slowly-migrated bands, respectively. (**B**–**H**) Changes of Conductivity (**B**,**E**), Ca^2+^ concentration (**C**,**F**), and K^+^ concentration (**D**,**G**,**H**) in the cell suspension treated with SGP7.0, UPW, HEPES7.0 or HEPES6.0 for 3 h measured every 30 min during UPW- or HEPES6.0-treatment. Plots based on triplicate means and polynomial approximation curves based on Microsoft Excel 2010 are expressed.

The bacterial chromosome is commonly circular double-stranded DNA which is packaged into smaller spaces through supercoiling [20]. Supercoiled, nicked circular, and linear forms of DNA are distinguishable by means of migration in agarose gel electrophoresis [21]. Genomic DNAs were isolated from OUMS1 cells treated with UPW, SGP7.0, HEPES6.0, or HEPES7.0 for 3 h and the status of the genomic DNAs was compared by migration in agarose gel. As shown in Figure 5A, major bands (indicated by arrowheads) obtained from the autolytic cells treated with UPW or HEPES6.0 migrated more slowly than those obtained from the vital cells in SGP7.0 or HEPES7.0 treatment. This major band probably reflects the relaxed-state of supercoiled DNA in the autolytic cells. It was noteworthy that an additional band (indicated by asterisks in Figure 5A) which migrated more slowly than the major band appeared in the autolytic cell preparation, suggesting that the genomic DNA was partially damaged and lapsed into the relaxed state. However, the possibility remains that relaxation of the genomic DNA may be a result of metabolism or gene expression in response to environmental changes, as reported previously [22].

Drastic damage of the plasma membrane in UPW- or HEPES6.0-treated cells led us to assume that the intracellular electrolytes could be leaked from the autolytic cells, resulting in change of conductivity and cation concentration in the cell suspension. Indeed, osmotic-shocked bacteria, even while remaining viable, were reported to release a number of cytoplasmic molecules including ions, metabolites, and certain proteins through damaged bacterial envelope [23]. Thus, changes of conductivity and Ca^2+^/K^+^ concentrations in the cell suspension were monitored during UPW- and HEPES6.0-treatments (Figure 5B–D), comparing with those in HEPES7.0-treated cells, because the latter cells remained alive for at least 3 h (Figure 2A,C). In UPW-treated cells, conductivity of the cell suspension increased after the onset of treatment, whereas in HEPES7.0-treated cells its value was almost steady (Figure 5B). Concentration of both K^+^ and Ca^2+^ increased 30–90 min after the onset of treatment (Figure 5C,D). Similar changes of conductivity and K^+^/Ca^2+^ concentration were observed in HEPES6.0 *versus* HEPES7.0-treated cells (Figure 5E–G). It was most likely that the electrolytes such as K^+^ and Ca^2+^ were leaked from the autolytic cells due to damage of plasma membrane, as imagined from the TEM observation (Figure 4C–F). As expected from results in Figure 3D, K^+^ leakage did not occur when CaCl_2_ was added to HEPES6.0 (Figure 5H).

### 3.6. Lysozyme-Inducing Autolysis of OUMS1 Due to Damage of Peptidoglycan

Several lines of evidence [8,9,10,11] showed that bacterial autolysis induced by osmotic or pH shock gave rise to damage of peptidoglycans and that it was suppressed by the presence of Mg^2+^ or Ca^2+^. To examine whether autolysis of OUMS1 cells could be associated with damage of peptidoglycan, these cells were incubated with lysozyme, an enzyme catalyzing the hydrolysis of 1,4-β-linkages between *N*-acetylmuramic acid and *N*-acetyl-d-glucosamine residues in peptidoglycans [24]. Incubation of OUMS1 cells with 100 to 400 μg/mL of lysozyme imposed autolysis within hours (see Supplementary Figure S1A). The treatment of cell suspension with HEPES7.0 containing 200 μg/mL lysozyme enhanced elevation of conductivity and Ca^2+^/K^+^ concentrations in the suspension, suggesting that damage of peptidoglycans provoked the release of electrolytes from the autolytic cells (see Supplementary Figure S1B–D).

Incubation with 200 μg/mL of lysozyme in the presence of 0.3–3 mM CaCl_2_ for hours led the cells to form colonies in the drop test (see Supplementary Figure S1E), indicating that the lysozyme-dependent autolysis was suppressed by Ca^2+^. The L/D stain (see Supplementary Figure S1F) and measurement of K^+^ concentration in the presence of 3 mM CaCl_2_ (see Supplementary Figure S1G) supported the above conclusion.

Wegner *et al.* [10] reported that HEPES buffer (pH 8.5)-inducing autolysis of *N. gonorrhoeae* cells was accompanied by the hydrolysis of peptidoglycan and was inhibited by the addition of Mg^2+^, Ca^2+^, Mn^2+^, and Ba^2+^. However, they found that the rate of peptidoglycan hydrolysis in HEPES was maximal at pH 8.5 and was similar in the presence or absence of Mg^2+^, concluding that divalent cation stabilization against autolysis could not be mediated by inhibition of peptidoglycan hydrolysis. Although the present experiments showed that the lysozyme-inducing autolysis was interfered by Ca^2+^, it remains uncertain whether the above acidic pH-dependent autolysis was attributable to damage of peptidoglycan. Further detailed experiments are required before making a conclusion. These lysozyme-related experiments led us to assume that globular outgrowth of outer plasma membrane in UPW- and HEPES6.0-treated cells (Figure 4E,F) could be attributable to damage of peptidoglycan.

### 3.7. Lysozyme-inducing Autolysis of OUMS1 Due to Damage of Peptidoglycan

Several lines of evidence [8,9,10,11] showed that bacterial autolysis induced by osmotic or pH shock gave rise to damage of peptidoglycans and that it was suppressed by the presence of Mg^2+^ or Ca^2+^. To examine whether autolysis of OUMS1 cells could be associated with damage of peptidoglycan, these cells were incubated with lysozyme, an enzyme catalyzing the hydrolysis of 1,4-β-linkages between *N*-acetylmuramic acid and *N*-acetyl-d-glucosamine residues in peptidoglycans [24]. Incubation of OUMS1 cells with 100 to 400 μg/mL of lysozyme imposed autolysis within hours (see Supplementary Figure S1A). The treatment of cell suspension with HEPES7.0 containing 200 μg/mL lysozyme enhanced elevation of conductivity and Ca^2+^/K^+^ concentrations in the suspension, suggesting that damage of peptidoglycans provoked the release of electrolytes from the autolytic cells (see Supplementary Figure S1B–D).

Incubation with 200 μg/mL of lysozyme in the presence of 0.3–3 mM CaCl_2_ for hours led the cells to form colonies in the drop test (see Supplementary Figure S1E), indicating that the lysozyme-dependent autolysis was suppressed by Ca^2+^. The L/D stain (see Supplementary Figure S1F) and measurement of K^+^ concentration in the presence of 3 mM CaCl_2_ (see Supplementary Figure S1G) supported the above conclusion.

Wegner *et al.* [10] reported that HEPES buffer (pH 8.5)-inducing autolysis of *N. gonorrhoeae* cells was accompanied by the hydrolysis of peptidoglycan and was inhibited by the addition of Mg^2+^, Ca^2+^, Mn^2+^, and Ba^2+^. However, they found that the rate of peptidoglycan hydrolysis in HEPES was maximal at pH 8.5 and was similar in the presence or absence of Mg^2+^, concluding that divalent cation stabilization against autolysis could not be mediated by inhibition of peptidoglycan hydrolysis. Although the present experiments showed that the lysozyme-inducing autolysis was blocked by Ca^2+^, it remains uncertain whether the above acidic pH-dependent autolysis was attributable to damage of peptidoglycan. Further detailed experiments are required before making a conclusion. These lysozyme-related experiments led us to assume that globular outgrowth of outer plasma membrane in UPW- and HEPES6.0-treated cells (Figure 4E,F) could be attributable to damage of peptidoglycan.

### 3.8. Autolysis of Leptothrix Discophora Strain SP-6 Cells Induced by the UPW Treatment

To examine whether the UPW-dependent autolysis occurs in other members of *Leptothrix*, SP-6 cells were subjected to the same UPW treatment. A similar autolytic effect was confirmed by the drop test and the L/D stain (see Supplementary Figure S2). However, it is too early to conclude that autolytic effects by UPW are common among the genus of *Leptothrix,* since the pH preference for growth is variable among species of *Leptothrix,* as Spring [2] noted: at pH 8.5 *L. cholodnii* and *L. mobilis* grow but *L. discophora* does not. Further investigations against several other species and/or strains are required to determine whether this type of autolysis is common in members of the genus *Leptothrix* including the habitant species of dentine caries [25].

The present overall results demonstrated the inducing effect of acidic pH on autolysis of OUMS1 and SP-6 cells and the suppressive effect of Ca^2+^ on the pH-dependent autolysis as similarly as those reported in several Gram-negative bacteria [8,10,11]. Our previous paper [1] reported that spontaneous autolysis of OUMS1 cells could be a major cause of hollowing of aged sheaths. However, we were unable to determine why and how the spontaneous autolysis occurred within the sheaths. The present results led us to assume that aqueous-phase cations, especially Ca^2+^, could be trapped by the sheaths, resulting in local changes of pH, conductivity, and cation concentrations within the sheaths. Such local environmental changes within sheaths may trigger the spontaneous autolysis of the nearby cells.

## 4. Conclusions

We previously reported that sheaths of *Leptothrix* sp. strain OUMS1 cultured in artificial media became hollow with aging due to spontaneous autolysis within the sheaths. To approach the mechanism of this autolysis more closely, we undertook to examine basic physiology of OUMS1 cells affected by the environmental conditions which promoted autolytic death of the cells, and in particular, the effects of UPW on the sudden environment changes which might affect the bacterial viability. Treatment of the cells with UPW or acidic buffers (pH 6.0) caused autolysis of the cells. Under these conditions, the plasma membrane and cytoplasm of cells were drastically damaged, resulting in leakage of intracellular electrolytes and relaxation of genomic DNA. The autolysis was suppressed by the presence of Ca^2+^. The hydrolysis of peptidoglycan by the lysozyme treatment similarly caused autolysis of the cells and was suppressed also by the presence of Ca^2+^. *Leptothrix discophora* strain SP-6 cells were also killed when suspended in UPW. *Leptothrix* may survive within a narrow pH range and changes of environmental ion concentration, in particular Ca^2+^, may trigger life- and death-crisis for this bacterium. Based on the present phenomenological observations with a few mechanistic insights, further physiological and some molecular level experiments with a non-sheath-forming *Leptothrix* strain as control are ongoing for clarifying what the cellular response may be in terms of changes in cell physiology, or how much the sheath helps to prevent autolysis.

## Supplementary Files

Supplementary File 1
