# Supplementary material for: Treatment of Leptothrix Cells with Ultrapure Water Poses a Threat to Their Viability
Source: Biology (Basel). 2015 Jan 27;4(1):50–66. doi: 10.3390/biology4010050 (PMC4381217; doi:10.3390/biology4010050)
Supplement: Supplementary File 1 [file biology-04-00050-s001.pdf]

Supplemental Material

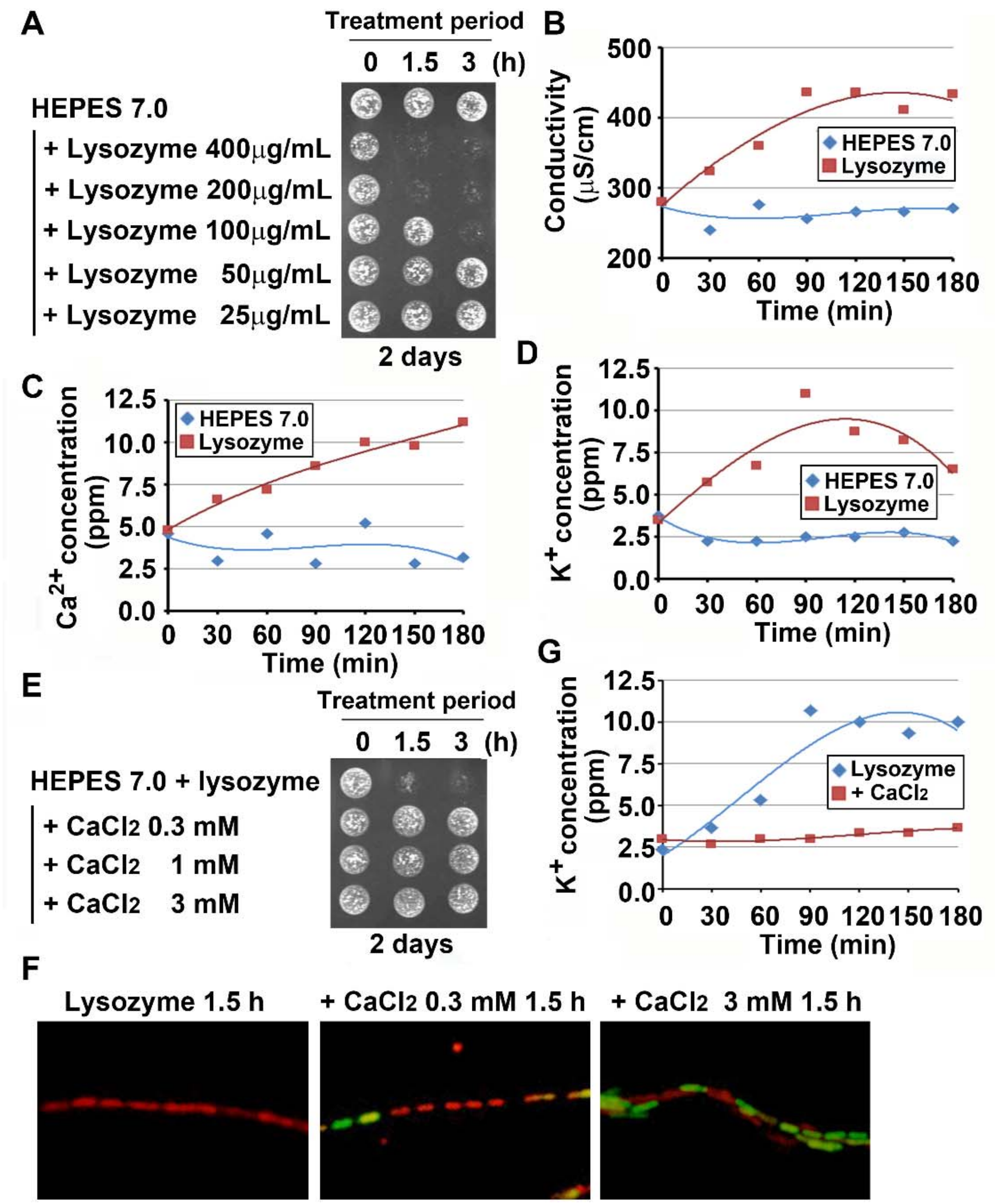

Figure S1. Autolysis induced by lysozyme treatment of OUMS1 cells due to damage of peptidoglycan. (**A**) Colony formation by the cells treated with HEPES7.0 added with lysozyme (0–400 μg/mL). Note no colony formation by the cells treated with HEPES7.0 containing 200 and 400 μg/mL for 1.5 or 3 h. (**B**) Changes of conductivity of cell suspensions during HEPES7.0 or UPW treatment. (**C**) Changes of Ca^{2+} concentration in cell suspension during HEPES7.0 or UPW treatment. (**D**) Changes of K^+ concentration in cell suspension during HEPES7.0 or UPW treatment. (**E**) Suppression of autolysis induced by HEPES7.0 containing lysozyme in the presence of $CaCl_2$ (**G**) Suppression of K^+ leakage by Ca^{2+} from the autolytic cells treated with HEPES7.0 containing lysozyme. (**F**) The L/D stain images of the cells treated with HEPES7.0 containing lysozyme and 0–3 mM $CaCl_2$.

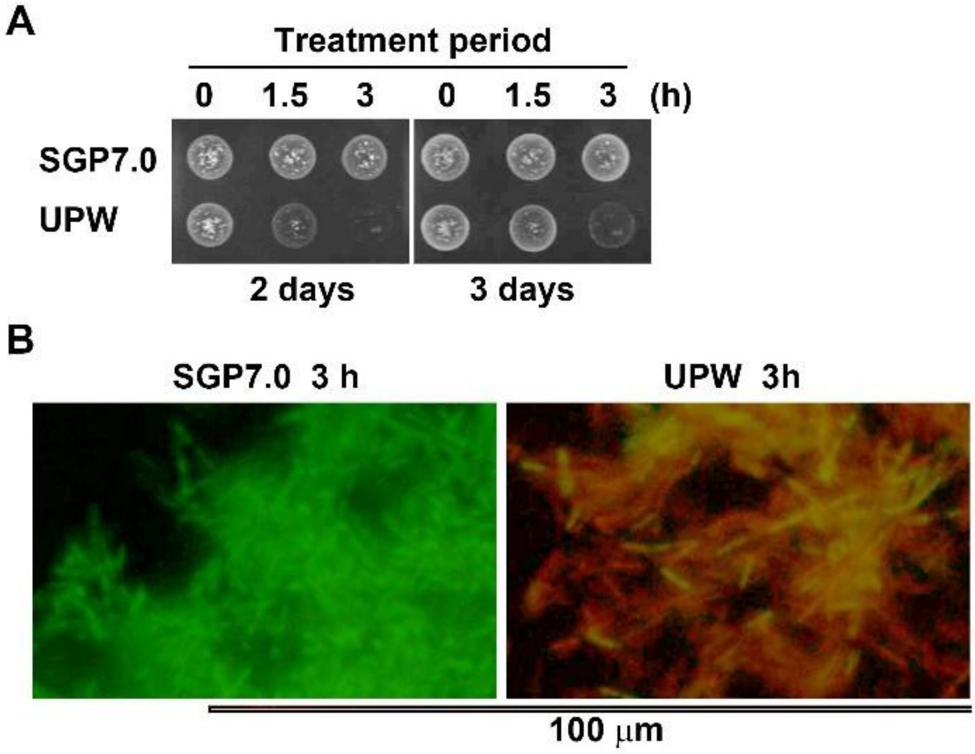

Figure S2. Autolysis of SP-6 cells induced by UPW treatment. (**A**) Colony formation by SP-6 cells treated with UPW or SGP7.0 for 0–3 h. Note that the 3 h UPW treatment did not allow the cells to form colony. (**B**) The L/D stain images of SP-6 cells treated with SGP7.0 or UPW for 3 h. Note that the latter treatment resulted in the death of all cells.
